# Ixazomib: An Oral Proteasome Inhibitor for the Treatment of Multiple Myeloma

**Published:** 2017-05-01

**Authors:** Krista G. Ramirez

**Affiliations:** Greenville Health System, Greer, South Carolina

## Abstract

The FDA approval of ixazomib for use in combination with lenalidomide and dexamethasone for patients with multiple myeloma who have received at least one prior therapy signalled the arrival of the first oral proteasome inhibitor, allowing advanced practitioners to provide multiple myeloma patients an exclusively oral triplet therapy.

Multiple myeloma (MM) is a hematologic malignancy characterized by the proliferation of plasma cells that produce monoclonal immunoglobulins, or paraproteins. Due to the rapid proliferation of these cancerous plasma cells, normal blood cells are "crowded out" of the bone marrow, and the immune response is compromised. Patients with MM commonly present with anemia, renal dysfunction, and skeletal fractures (Rajkumar, 2016).

Multiple myeloma is the second most common hematologic malignancy, with 30,300 new diagnoses and 12,650 deaths estimated in 2016 (Siegel, Miller, & Jemal, 2016). Since the introduction of immunomodulatory agents and proteasome inhibitors into MM treatment regimens, overall survival (OS) has increased by twofold to threefold (Dou & Zonder, 2014). The 5-year survival rate from the time of diagnosis is 48.5% (Siegel et al., 2016).

According to the 2017 National Comprehensive Cancer Network (NCCN) Guidelines for MM, three-drug regimens are superior to doublet therapy in response rate and depth of response. The International Myeloma Working Group consensus on the treatment of MM with high-risk cytogenetics is that newly diagnosed patients should be treated with triplet therapy, including a proteasome inhibitor with lenalidomide (Revlimid) or pomalidomide (Pomalyst) and dexamethasone (2016).

## MECHANISM OF ACTION

Proteasomes are the cellular mechanism by which most proteins are broken down and cleared from the cell. Proteins destined for degradation are tagged with regulatory proteins called ubiquitin, which are then recognized by the proteasome as a signal to break down the protein. This intracellular protein breakdown process is the ubiquitin-proteasome system (Dou & Zonder, 2014).

In the treatment of MM, proteasome inhibitors block the plasma cell proteasomes from breaking down the ubiquitin-tagged paraproteins. The accumulation of paraproteins ultimately results in myeloma cell apoptosis (Dou & Zonder, 2014). Malignant cells possess higher levels of proteasome activity as compared with healthy cells, which makes the proteasome a rational target for drug development (Moreau et al., 2012).

## DRUG DEVELOPMENT AND HISTORY

Bortezomib (Velcade), the first proteasome inhibitor, was approved in 2003 (Millennium Pharmaceuticals, 2015a). Although bortezomib significantly improved outcomes for MM patients, some resistance was observed. This prompted the development and subsequent approval of a second-generation proteasome inhibitor, carfilzomib (Kyprolis), in 2012. Carfilzomib is an irreversible proteasome inhibitor that exhibited a decreased incidence of peripheral neuropathy in clinical studies, a notable class effect of proteasome inhibitors (Onyx Pharmaceuticals, 2016).

On November 20, 2015, the US Food and Drug Administration (FDA) granted approval to ixazomib (Ninlaro) for use in combination with lenalidomide and dexamethasone for patients with MM who have received at least one prior therapy (FDA, 2015). Until the approval of ixazomib, proteasome inhibitors were only available as intravenous infusions or subcutaneous injections. Ixazomib is the first oral proteasome inhibitor, allowing the advanced practitioner to provide MM patients an exclusively oral triplet therapy.

## CLINICAL TRIAL

Efficacy of ixazomib was demonstrated in a phase III randomized, double-blind, placebo-controlled, multicenter study conducted by Moreau et al. (2016). Patients (n = 722) with relapsed and/or refractory MM were randomized to receive either ixazomib, lenalidomide, and dexamethasone (n = 362) or placebo, lenalidomide, and dexamethasone (n = 360). 

Randomization was stratified based on prior proteasome inhibitor therapy, number of lines of prior therapy, and severity of disease. A total of 70% of patients had previous exposure to a proteasome inhibitor, 69% had received bortezomib, 45% had received thalidomide, and 12% had previously received lenalidomide.

Ixazomib demonstrated a statistically significant (p = .012) progression-free survival (PFS) advantage, with a median PFS of 20.6 months vs. 14.7 months in the control arm. An OS benefit was not demonstrated. The PFS benefit was consistent among all predetermined subgroups, including patients with high-risk cytogenetic abnormalities, International Staging System III disease, age greater than 75 years, and receipt of two or three prior therapies (Moreau et al., 2016). Additional phase III trials are ongoing to evaluate the potential role of ixazomib in newly diagnosed, previously untreated patients with MM (Takeda Pharmaceutical Company Ltd., 2015).

## ADVERSE EFFECTS

Among patients receiving ixazomib in the trial conducted by Moreau et al. (2016), the most common adverse effects (those occurring in ≥ 20% of patients) were diarrhea, constipation, nausea, vomiting, fluid retention, peripheral neuropathy, back pain, thrombocytopenia, and rash.

The most common severe side effects experienced by at least 2% of study participants were thrombocytopenia and diarrhea (Millennium Pharmaceuticals, Inc., 2015b). While peripheral neuropathy is a familiar class effect, the incidence is notably reduced in patients receiving ixazomib as compared with bortezomib (Dou & Zonder, 2014). Moreau et al. reported in their 2016 study that ≥ grade 3 neuropathy occurred in 2% of patients in both treatment groups, whereas Durie et al. (2015) reported ≥ grade 3 neuropathy in 24% of patients in the bortezomib, lenalidomide, and dexamethasone arm vs. 5% in the lenalidomide and dexamethasone arm.

Per the 2017 NCCN guidelines for the prevention and treatment of cancer-related infections, treatment with proteasome inhibitors warrants antiviral prophylaxis due to the risk of reactivation of herpes simplex virus (HSV) and varicella zoster virus (VZV). Patients should be prescribed an appropriate antiviral prophylactic during active treatment with ixazomib and throughout periods of neutropenia (NCCN, 2017b).

## DOSING AND ADMINISTRATION

Ixazomib is approved for use in combination with lenalidomide and dexamethasone (IRd). The recommended ixazomib starting dose is 4 mg administered weekly on days 1, 8, and 15 of a 28-day cycle, detailed in Table 1. A reduced starting dose of 3 mg is recommended for patients with moderate to severe hepatic impairment, severe renal impairment, or end-stage renal disease on dialysis (Table 2). Ixazomib is nondialyzable (Millennium Pharmaceuticals, Inc., 2015b).

**Table 1:**
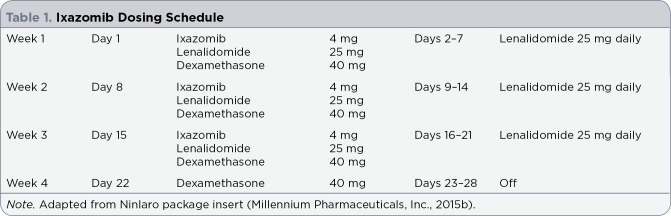
Ixazomib Dosing Schedule

**Table 2:**
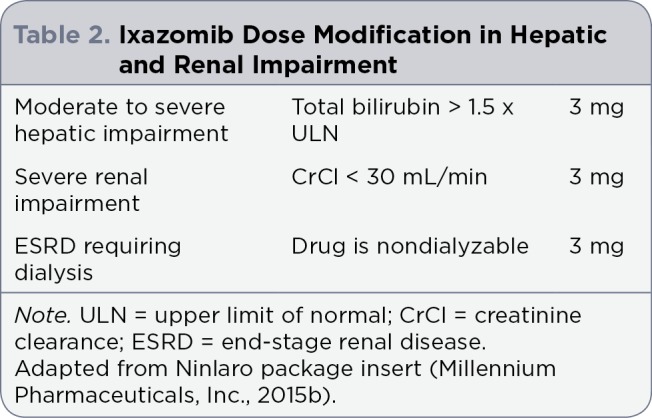
Ixazomib Dose Modification in Hepatic and Renal Impairment

Patients should be instructed to take ixazomib with a full glass of water 1 hour before or 2 hours after a meal. The advanced practitioner should discuss separation of ixazomib and dexamethasone doses with patients, as dexamethasone should be taken with food. Missed doses of ixazomib should only be made up if there are ≥ 72 hours remaining before the next dose is due (Millennium Pharmaceuticals, Inc., 2015b).

## PATIENT MONITORING AND COUNSELING

The advanced practitioner should obtain a complete blood cell count at least monthly to monitor both platelets and absolute neutrophil count (ANC); platelets and ANC should recover to 75,000/mm³ and 1,000/mm³, respectively, prior to initiating a new cycle. Platelet nadir occurs between days 12 and 21 of a 28-day cycle. Thrombocytopenia and neutropenia can be managed with dose reductions as outlined in Table 3. It is also recommended to periodically monitor liver function throughout treatment (Millennium Pharmaceuticals, Inc., 2015b).

**Table 3:**
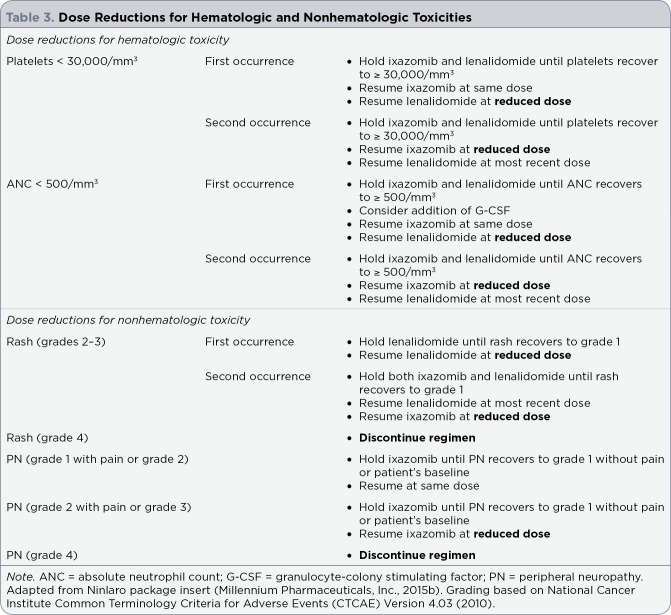
INFORTable 3. Dose Reductions for Hematologic and Nonhematologic Toxicities

Ixazomib is dispensed in blister packs and should not be removed until it is time to take the dose. Many patients find pill boxes particularly helpful, especially with complicated dosing regimens such as this one; therefore, it is important to educate patients on proper medication storage.

Patients may find it helpful to keep a journal to document the progression and severity of adverse effects. This documentation can help the advanced practitioner to objectively assess the need for nonhematologic dose reductions (Table 3). Patients should be instructed to contact the office if they notice unusual bleeding or bruising, fever, vomiting or diarrhea that persists despite treatment, excessive weight gain or swelling, or yellowing of the skin or eyes (Millennium Pharmaceuticals, Inc., 2015b).

Performing thorough medication reconciliation prior to initiating therapy is crucial to ensure safe administration of treatment. The use of over-the-counter and herbal medications should be evaluated with the same scrutiny as prescription medications. Strong cytochrome P450 (CYP) 3A inducers such as rifampin, phenytoin, carbamazepine, and St. John’s wort should be avoided (Millennium Pharmaceuticals, Inc., 2015b).

## SUMMARY

Proteasome inhibitors have contributed significantly to the increased survival and improved outcomes in MM patients. With the recent approval of ixazomib, the therapeutic benefits of proteasome inhibitors are no longer restricted to parenteral formulations. The advanced practitioner can provide disease and drug education, side-effect management, timely monitoring, and routine follow-up to ensure patients gain the most benefit from this novel treatment option.
